# The Pan-Sirtuin Inhibitor MC2494 Regulates Mitochondrial Function in a Leukemia Cell Line

**DOI:** 10.3389/fonc.2020.00820

**Published:** 2020-05-21

**Authors:** Vincenzo Carafa, Rosita Russo, Laura Della Torre, Francesca Cuomo, Carmela Dell'Aversana, Federica Sarno, Giulia Sgueglia, Marzia Di Donato, Dante Rotili, Antonello Mai, Angela Nebbioso, Gilda Cobellis, Angela Chambery, Lucia Altucci

**Affiliations:** ^1^Dipartimento di Medicina di Precisione, Università degli Studi della Campania “Luigi Vanvitelli”, Naples, Italy; ^2^Dipartimento di Scienze e Tecnologie Ambientali Biologiche e Farmaceutiche, Università degli Studi della Campania “Luigi Vanvitelli”, Caserta, Italy; ^3^Institute Experimental Endocrinology and Oncology “Gaetano Salvatore” (IEOS)- National Research Council (CNR), Naples, Italy; ^4^Dipartimento di Chimica e Tecnologie del Farmaco, “Sapienza” Università di Roma, Rome, Italy

**Keywords:** sirtuins, epigenetic, mitochondria, cancer, leukemia, energy production, proteomic analysis

## Abstract

The involvement of sirtuins (SIRTs) in modulating metabolic and stress response pathways is attracting growing scientific interest. Some SIRT family members are located in mitochondria, dynamic organelles that perform several crucial functions essential for eukaryotic life. Mitochondrial dysfunction has emerged as having a key role in a number of human diseases, including cancer. Here, we investigated mitochondrial damage resulting from treatment with a recently characterized pan-SIRT inhibitor, MC2494. MC2494 was able to block mitochondrial biogenesis and function in terms of ATP synthesis and energy metabolism, suggesting that it might orchestrate cell response to metabolic stress and thereby interfere with cancer promotion and progression. Targeting mitochondrial function could thus be considered a potential anticancer strategy for use in clinical therapy.

## Introduction

Sirtuins (SIRTs) are a group of highly conserved proteins belonging to the class III histone deacetylase family of enzymes. The seven mammalian SIRTs display distinct protein structures, subcellular locations, and unique functional properties (1). Of the seven members, SIRT3, SIRT4, and SIRT5 predominantly reside in the mitochondria, where they regulate energy metabolism in response to oxidative stress (2). SIRTs are the principal class of enzymes that act as metabolic sensors. Because of their involvement in modulating several metabolic and stress response pathways, SIRTs have been attracting considerable scientific interest (3) SIRTs are currently the subject of major investigations sparked by the long-standing debate over their dual function in cancer biology as either tumor promoters or tumor suppressors (4–6). Abnormal energy metabolism resulting from reprogrammed metabolic pathways is one of the hallmarks of cancer, and SIRTs are essential regulators of these networks, acting via several and different pathways (7).

Warburg demonstrated for the first time in 1927 that cancer cells undergo anaerobic glycolysis, producing lactate as a consequence of high metabolic demands, decreasing mitochondrial glucose oxidation, and increasing fatty acid metabolism, which in turn increase reactive oxygen species (ROS) production. In 2011, the list of cancer hallmarks originally described by Hanahan and Weinberg was in fact extended to include reprogramming energy metabolism (7).

Mitochondria are cytoplasmic organelles that play a key role in energy production, metabolism, and cell death as well as in other cellular processes (3, 8, 9). They perform many critical cellular functions, including maintenance of metabolic homeostasis through the process of oxidative phosphorylation (OXPHOS) (10) and generation of adenosine triphosphate (ATP) (11). Several proteins are involved in the induction of mitochondrial biogenesis, also regulating many metabolic processes, such as thermogenesis, respiration, and gluconeogenesis (12, 13), underlining the central role of mitochondria as regulators of metabolism. Changes in mitochondrial number and loss of function are implicated in many diseases, including cancer (14, 15). Although the link between mitochondrial dysfunction and cancer initiation is well-established, the underlying molecular mechanisms are still poorly understood.

Several studies describe the correlation between tumourigenesis and alteration of cellular metabolism, triggered in response to the energetic demands of cancer cells necessary to sustain increased cell proliferation and invasion (16). Given dynamic role of mitochondria, many studies are focusing on the involvement of mitochondria in cancer development and progression. SIRTs are able to modulate mitochondrial function via regulation of ATP production, metabolism, apoptosis, and cell signaling.

Here, we investigated mitochondrial regulation by a recently characterized pan-SIRT inhibitor, MC2494. MC2494 is known to be able to induce tumor-selective cell death pathways involving mitochondria in terms of intrinsic caspase activation, reactive oxygen species (ROS) production, and dissipation of mitochondrial membrane potential (MMP) (17). We found that MC2494 has a general effect on mitochondrial functions and may therefore orchestrate cell response to metabolic stress, interfering with tumourigenesis. Our findings support targeting mitochondrial function as a potential anticancer strategy for use in clinical therapy.

## Materials and Methods

### Reagents and Cell Lines

All chemicals, including tosyl phenylalanyl chloromethyl ketone (TPCK)-treated trypsin, were from Thermo Scientific, unless otherwise stated. Acetonitrile (CH_3_CN, #15611400, Honeywell Riedel-de Haen), formic acid (FA, #10627431) and LC-MS grade water (#15651400, Honeywell Riedel-de Haen) were from Fisher Scientific. MC2494 was prepared as previously reported (17). Antibodies: PGC1α (#ab191838), PGC1β (#ab176328), and SOD2 (#ab13533) were from Abcam and GAPDH was from Santa Cruz (#sc-47724). Cell lines: U937 (#ACC5) human myeloid leukemia cells were purchased from DSMZ and MCF7 (#ICLCHTL95021) breast cancer cells from Cell Bank Interlab Cell Line Collection. U937 and MCF7 cells were propagated in RPMI (Euroclone #ECB9006) and DMEM (Euroclone #ECB7501), respectively, with 10% fetal bovine serum (FBS; Gibco #10270), 2 mM L-glutamine (Euroclone #ECB3000D), and antibiotics (100 U/mL penicillin, 100 μg/mL streptomycin, Euroclone #ECB3001D, and 250 ng/mL amphotericin-B; Euroclone #ECM0009). All cell lines were grown at 37°C with 5% CO_2_, and were then tested, authenticated, and used for 10–20 passages. Mycoplasma contamination was checked using EZ-PCR Mycoplasma Test Kit (Biological Industries #20-700-20).

### MTT and WST-1 Assays

U937 cell viability was determined using 3-(4,5-dimethylthiazol-2-yl)-2,5-diphenyltetrazolium bromide (MTT) assay (Sigma-Aldrich #M5655), and sodium salt of 4-[3-(4iodophenyl)-2-(4-nitrophenyl)-2H-5-tetrazolio]-1,3-benzene disulfonate (WST-1) assay (Roche #11644807001). For MTT assay, 2 × 10^5^ cells/well were plated in a 48-well plate and treated, in triplicate, with MC2494 at 50, 25, 12.5, 6.25, 3.125 1.625, and 0.81 μM concentration for 24 h, and with DMSO (Sigma-Aldrich #D4540) used as a control. MTT water solution was added to each well at 0.5 mg/mL. After 4 h, the plate was centrifuged at 1,000 × g for 5 min and the supernatant was removed. The purple formazan crystals were dissolved in iso-propanol (Sigma #33539) and stirred for 15 min. Absorbance was read at a wavelength of 570 nm with a TECAN M-200 reader (TECAN). IC_50_ values were calculated using GraphPad Prism7 software. For WST-1 assay, 1 × 10^4^ cells/well were plated in a 96-well plate and treated with MC2494 at 50 μM concentration for different times (0.5–24 h). After 1.5 h of incubation, the plate was immediately read at 460 nm with a TECAN M-200 reader (TECAN).

### Western Blot

Whole cell extract was obtained after suspension in lysis buffer (50 mM Tris-HCl pH 7.4, 150 mM NaCl, 1% NP40, 10 mM NaF, 1 mM PMSF, and protease inhibitor cocktail), for 15 min at 4°C. After centrifugation, protein concentration was determined by Bradford assay (Bio-Rad #5000006). A total of 50 μg of proteins was loaded on 10–15% polyacrylamide gels and transferred onto a nitrocellulose membrane using Trans-Blot Turbo Transfer System (Bio-Rad #1704159) according to the manufacturer's instructions. After a step of blocking in 5% milk in Tris-buffered saline with Tween (TBST; 10 mM Tris pH 8.0, 150 mM NaCl, 0.5% Tween 20), the membrane was washed and incubated with antibodies. Detection was performed with an enhanced chemiluminescence system (Amersham Biosciences) according to the manufacturer's protocol.

### ATP and ATPase Assays

A colorimetric method was used to evaluate ATP (Elabscience, E-BC-K157-S) and ATPase (Elabscience, E-BC-K108) content. Assays were carried out following the manufacturer's instructions. Briefly, 2 mL of U937 suspension cells at a confluence of 2 × 10^5^ cells/mL were treated with MC2494 at a concentration of 25 μM in a 24-well plate for different times (0.5–3 h). At the end of the reaction, absorbance was read using a TECAN M-200 reader (TECAN) at 636 nm. Each experiment was performed in biological triplicates and values expressed as mean ± SD.

### Calcium Assay

To evaluate calcium content was used a colorimetric assay (Elabscience, E-BC-K103-M). Assay was carried out following the manufacturer's instructions. Briefly, 2 mL of U937 cells at a confluence of 2 × 10^5^ cells/mL were treated with MC2494 at a concentration of 25 μM for 24 h. At the end of the reaction, absorbance was read using a TECAN M-200 reader (TECAN) at 610 nm. Each experiment was performed in biological triplicates and values expressed as mean ± SD.

### Mitochondria Content

U937 cells were treated with MC2494 for different times (0.5–24 h). After treatment, MitoTracker Orange dye (Invitrogen #M7510) at a final concentration of 10 μM was added for 30 min. After 2 washes in PBS, cellular pellets were suspended in PBS containing propidium iodide (0.5 μg/mL) and analyzed with FACS Calibur (BD Biosciences). Data analysis was performed using CellQuest software (BD Biosciences).

### Mitochondria Labeling

Labeling solution prepared with MitoTracker Orange dye (Invitrogen) at a final concentration of 1 μM was added to medium and cells were incubated for 45 min. After 3 washes with PBS, cells were fixed in 4% paraformaldehyde (Sigma-Aldrich #P6148) for 20 min. After 2 washes with PBS, nuclei were stained using Hoechst 33258 (Sigma-Aldrich #94403) at a final concentration of 1 μg/mL. Finally, coverslips were inverted and mounted in Mowiol (Calbiochem #475904100). Fields were observed using a DMBL Leica fluorescence microscope equipped with HCX PL Fluotar Apo 63X oil objectives. Images representative of 3 different experiments were captured with a DC480 camera (Leica) and acquired using Application Suite (Leica) software.

### Immunofluorescence Microscopy

U937 cells were cultured and grown on coverslips in 12-well plates. After MC2494 treatment, cells were fixed in methanol for 10 min at −20°C and blocked with BSA (2%) in TBST for 1 h. Blocked cells were stained with anti-PGC1α (Abcam #ab191838; ab#54481) diluted 1:100 in PBS overnight. The day after, coverslips were washed 3 times with PBS-BSA and incubated with anti-rabbit Texas Red-conjugated secondary antibody (diluted 1:400; Jackson Laboratories) for 1 h at room temperature. Nuclei were counterstained with DAPI (10 μg/mL; Molecular Probes #D1306), extensively washed with PBS-BSA, and mounted in Vectashield mounting medium (Vector Laboratories #H-1000-10). Fields were observed using a DMBL Leica fluorescence microscope equipped with HCX PL Fluotar Apo 63X oil objectives. Images representative of 3 different experiments were acquired and captured with a DC480 camera (Leica) using Application Suite (Leica) software.

### Cellular Energetics: Mitochondrial Stress and ATP Production

Cellular energetics was assessed in live cells using Seahorse XF24 Analyzer (Agilent Technologies). The oxygen consumption rate (OCR) and extracellular acidification rate (ECAR), key indicators of mitochondrial respiration and glycolysis, respectively, were determined with Seahorse XF Cell Mito Stress Test Kit (Agilent Technologies #103015) according to the manufacturer's instructions. Briefly, 3 × 10^5^ cells/well of MCF7 and U937 cells were plated on a Seahorse XF24 cell culture microplate and treated with 25 μM MC2494 for 30 min, 1 and 3 h at 37°C without CO_2_. Subsequently, the plate was loaded into the Seahorse XF24 Analyzer, and the OCR and ECAR were measured during sequential addition of 1 μM oligomycin A, 1 μM carbonylcyanide m-chlorophenylhydrazone (FCCP), 1 μM antimycin A and rotenone plus 0.5 μM antimycin A. ATP production rate was measured and quantified with Seahorse XF Real-Time ATP Assay Kit (Agilent Technologies #103592) according to the manufacturer's protocol. This protocol is similar to that of the Cell Mito Stress Test described above, except that ATP was measured during sequential addition of 5 μM oligomycin A and rotenone plus 15 μM antimycin A.

Data analysis was performed according to the manufacturer's indications. Experiments were performed in triplicate and repeated three times. Bars indicate mean ± SD. Statistical analyses were carried out using Student's *t*-test: ^*^p < 0.05; ^**^p < 0.01; and ^***^p < 0.001.

### Sample Preparation for Proteomic Analysis

For proteomic analysis, MC2494-treated with MC2494 at 50 μM and untreated U937 cells were lysed in ice-cold lysis buffer (100 mM triethylammonium bicarbonate [TEAB, #90114, Thermo Scientific], 1% SDS) and disrupted by two cycles of sonication at 20% amplitude for 30 sec on ice. Lysates were cleared by centrifugation at 16,000 × g for 15 min at 4°C. Supernatants were transferred into new tubes and treated with 1 unit of RQ1 DNase (#M6101, Promega) for 1 h at room temperature. Protein concentration was determined using Pierce BCA Protein Assay Kit (#23225, Thermo Scientific). For each condition, equal amounts of proteins (100 μg in 100 μL of 100 mM TEAB) were reduced with 10 mM tris-(2-carboxyethyl)-phosphine (TCEP part of TMT 10plex kit, #90113, Thermo Scientific) for 1 h at 55°C and alkylated with 18 mM iodoacetamide (part of TMT 10plex kit, #90113, Thermo Scientific) by incubating samples for 30 min at room temperature in the dark. Proteins were then precipitated overnight by adding 6 volumes of pre-chilled acetone (#15623200, Honeywell Riedel-de Haen, Fisher Scientific). Following centrifugation at 8,000 × g for 10 min at 4°C, protein pellets were resuspended in 100 μL of 100 mM TEAB, digested with trypsin (#90057, Thermo Scientific), and labeled with the following tandem mass tag (TMT, TMT 10plex kit, #90113, Thermo Scientific) isobaric tags as described elsewhere (18, 19) using 126 and 127N tags for MC2494-treated and untreated U937 cells, respectively. TMT-labeled samples were then mixed and diluted in 2% TFA (#28904, Thermo Scientific) to a final concentration of 0.5 μg/μL for liquid chromatography coupled to tandem mass spectrometry (LC-MS/MS) analysis.

### High-Resolution NanoLC-Tandem Mass Spectrometry

Aliquots of TMT-labeled peptides (2.5 μg) were analyzed by high-resolution nanoscale liquid chromatography coupled to tandem mass spectrometry (nanoLC-MS/MS) using a Q-Exactive Orbitrap mass spectrometer equipped with an EASY-Spray nanoelectrospray ion source (Thermo Scientific) coupled to a Dionex UltiMate 3000RSLC nano system (Thermo Scientific), as previously reported (19).

### Protein Identification and Quantitation

For data processing, the acquired raw files were analyzed with Thermo Scientific Proteome Discoverer 2.1 software (Thermo Fisher Scientific) using the SEQUEST HT search engine. The higher-energy collisional dissociation MS/MS spectra were searched against the *Homo sapiens* database (release 2019_11, 20380 entries) assuming trypsin (Full) as digestion enzyme and two allowed number of missed cleavage sites. Mass tolerances were set to 10 ppm and 0.02 Da for precursor and fragment ions, respectively. Oxidation of methionine (+15.995 Da) was set as dynamic modification. Carbamidomethylation of cysteine (+57.021 Da) and the TMT label on lysines and the N-terminus (229.1629) were set as static modifications. False discovery rates (FDRs) for peptide spectral matches were calculated and filtered using the Percolator node in Proteome Discoverer run with the following settings: Maximum Delta Cn 0.05, a strict target FDR of 0.01, a relaxed target FDR of 0.05, and validation based on q-value. Protein identifications were accepted when the protein FDR was below 1% and when present in at least two out of three replicate injections with at least two peptides. The list of U937 mitochondrial proteins identified by MS/MS was generated by including the subset of mitochondrial protein sequences from UniprotKB Swiss-prot database, selected based on the “Mitochondrion” term of the “Cellular component” gene ontology (GO) category.

### Bioinformatics Analysis

Functional enrichment based on GO categories was performed using FunRich open access software (http://funrich.org/index.html). The biological process enrichment network of identified mitochondrial proteins was constructed using the Network Analyst platform (https://www.networkanalyst.ca). A GOChord plot of selected GO categories extracted with the g:GOSt tool of the g:Profiler toolset (https://biit.cs.ut.ee/gprofiler/gost) was drawn using the GOplot package v1.0.2 of the RStudio v1.2.1335 environment for R (http://www.R-project.org).

### Statistical Analysis

Results are presented as the mean of 3 independent experiments ± SD. All data were analyzed using GraphPad Prism 6.0 software (GraphPad Software), and significance was determined by applying Student's *t*-test, one-way analysis of variance (ANOVA), and Dunnett's multiple-comparison test. Values with a *p* < 0.05 were considered significant.

## Results

### MC2494 Reduces Cell Proliferation and Metabolic Viability

**Figure 1:**
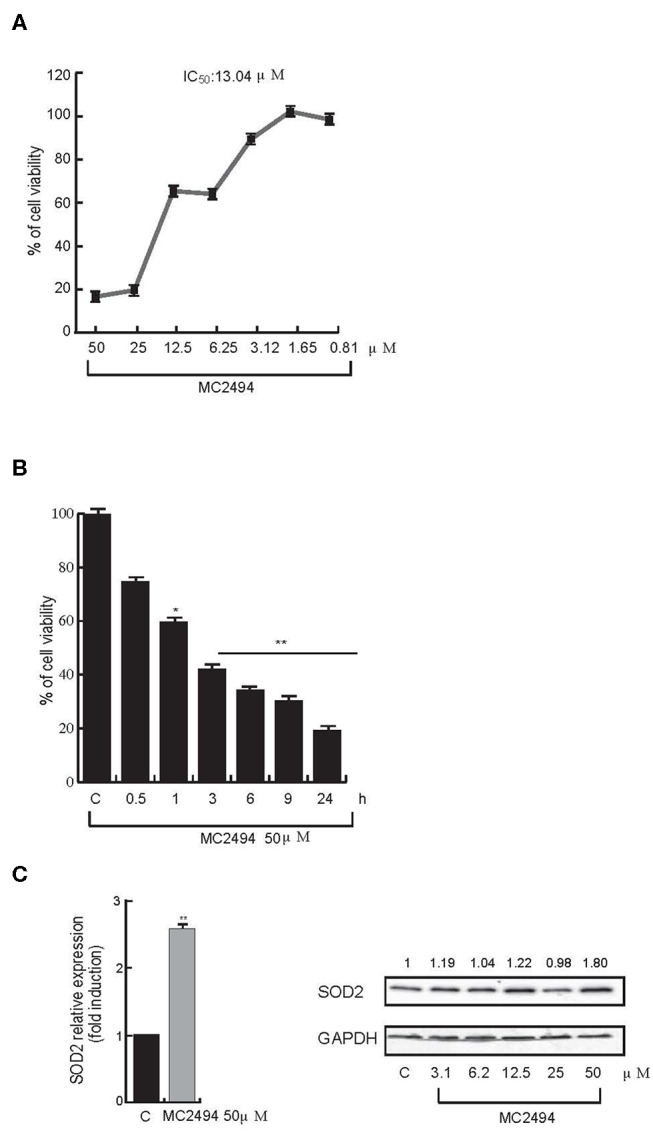
**(A)** Cell proliferation rate MTT assay. U937 cells were treated at indicated concentrations. IC_50_ value was calculated by GraphPad Prism 7. **(B)** Cell proliferation rate WST-1 assay. U937 cells were treated at 50 μM concentration for indicated times. **(C)** mRNA evaluation of *SOD2* (*Left panel*) and western blot analysis for SOD2 (*Right panel*). U937 cells were treated with MC2494 for 24 h at the indicated concentrations. Numbers on Western blot indicate the results of densitometry analysis, performed using the Image J Gel Analysis tool. RT-PCR graphs show the mean of at least two independent experiments with error bars indicating standard deviation. Values are mean ± standard deviation (*SD*) of biological triplicates. ^**^p ≤ 0.01, ^*^p ≤ 0.05 vs. control cells.

We previously reported that the novel pan-SIRT inhibitor MC2494 alters histone acetyltransferase/SIRT equilibrium in RIPK1 complex, blocking cell proliferation, and activating necroptotic cell death pathway (17, 20). Interestingly, we also observed a strong dissipation of MMP, leading to oxidative stress with substantial ROS release, upon MC2494 treatment (17), suggesting a possible link between MC2494-mediated cellular effects and mitochondrial dysfunction. To better understand the role of MC2494 in cellular homeostasis processes and mitochondrial involvement, we first investigated metabolic viability, which indirectly correlates with inhibition of cell proliferation. U937 cells were treated with MC2494 for 24 h at different concentrations and we observed a dose-dependent reduction of MTT to formazan product, indicating a decrease in metabolic viability and cell proliferation already after induction with MC2494 at 6.25 μM concentration, with an IC_50_ of 13.04 μM (Figure 1A). To further investigate this finding, inhibition of cell viability was also evaluated using water-soluble tetrazolium, the sodium salt of WST-1. U937 cells were treated with MC2494 at a fixed dose of 50 μM for different times of induction (0.5–24 h) and a significant time-dependent reduction of WST-1 to formazan product was observed already after 1 h of treatment, consistent with MTT results (Figure 1B).

Since the low rate of WST-1 formazan formation is linked to ROS activation, we investigated whether MC2494 treatment could increase expression of the mitochondrial dismutase gene *SOD2* as a cell defense mechanism against the toxicity of ROS. Quantitative RT-PCR analysis showed an increase in mitochondrial *SOD2* expression after 24 h of MC2494 treatment. These results were further confirmed by SOD2 western blot analysis following MC2494 treatment at different concentrations in the range 3.1–50 μM, showing an increase of SOD2 protein levels of about two-fold at the highest concentration tested (Figure 1C). Taken together these data support a mitochondrial involvement in MC2494-induced pathways.

### Mitochondrial Proteomic Analysis by High-Resolution NanoLC-MS/MS of MC2494-Treated U937 Cells

To investigate the effects of treatment with MC2494 on overall mitochondrial protein profile, we used the U937 cell line, shown to be a valuable preclinical model for investigating MC2494 effects on proliferation and caspase-8-dependent apoptosis (17).

**Figure 2:**
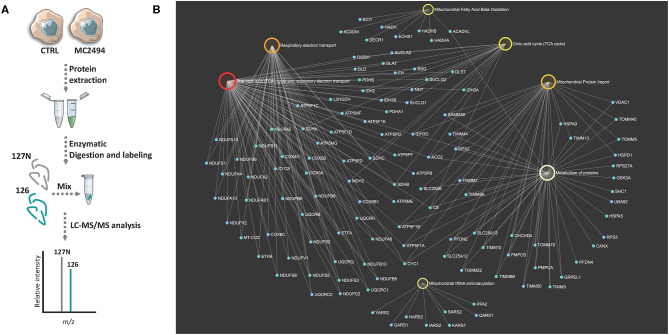
**(A)** Schematic workflow used for TMT-based quantitative analysis of mitochondrial proteome of MC2494-treated vs. untreated U937 cells. **(B)** Bipartite view of enrichment network including selected significant Gene Ontology (GO) categories and related identified mitochondrial proteins. Up- and down-regulated nodes are depicted in red and green, respectively, based on their log_2_ fold change values. Nodes are colored according to their *p*-value. The size of the node corresponds to the number of proteins in the list mapped on the specific term. The smaller nodes in light blue correspond to individual proteins/genes.

**Figure 3:**
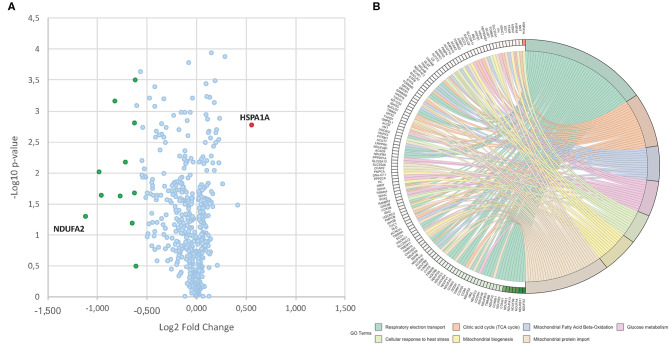
**(A)** Volcano plot obtained from TMT-based quantitative proteomic analysis of MC2494-treated vs. untreated U937 cells. Each point represents the difference in expression (log_2_ fold change) between MC2494-treated vs. untreated samples plotted against the –log_10_ *p*-value. Identified proteins with no changes in their regulation level are in light blue. Up- and down-regulated proteins (0.6≥ fold change ≥1.5) are shown in red and green, respectively. **(B)** GOChord plot showing relationships between selected representative biological process GO terms and related proteins identified by nanoLC-MS/MS analysis. The red and green color scale within the plot refers to protein log_2_ fold change values.

To identify differentially expressed proteins upon 24 h of induction with MC2494 at 50 μM, a comparative proteomic analysis was performed on treated and untreated U937 cells. A quantitative proteomic approach based on TMT isobaric labeling and nano-liquid chromatography coupled with high-resolution MS/MS analysis was adopted. A schematic workflow of the sample preparation and labeling procedure is shown in Figure 2A. About 80% of unique peptides was used for protein quantification, guaranteeing the high efficiency of peptide labeling (Supplementary Figures 1A,B). By MS/MS, we identified and quantified 500 non-redundant mitochondrial proteins with more than one unique peptide in at least two out of three injections (Supplementary Table 1). Enrichment analysis performed for the biological process GO category (Supplementary Figure 2A) revealed that a significant number of identified proteins are involved in processes related to energy pathways (38%/16%), metabolism/protein metabolism (38%/16%), and apoptosis (3%). Proteins related to fatty acid metabolism (0.6%), electron transport (0.4%), and mitochondrion organization and biogenesis (0.2) were also identified. The most represented terms within the molecular function GO category (Supplementary Figure 2B) were those of proteins with catalytic activity (16%) and oxidoreductase activity (8%). In addition, the enrichment network analysis performed on identified proteins against the Reactome database revealed a significant enrichment of proteins involved in mitochondrial respiratory electron transport, citric acid cycle, fatty acid beta-oxidation, protein metabolism, and tRNA aminoacylation (Figure 2B), showing the high-throughput potential of proteomic analysis. A very small fraction (about 2.4%) of identified proteins was found differentially expressed (0.6 ≥fold change ≥1.5) in MC2494-treated compared to untreated U937 cells (Figure 3A). A specific subset of down-regulated proteins was associated with respiratory electron transport and related pathways (Figure 3B and Supplementary Figure 3), including several subunits of the NADH dehydrogenase (ubiquinone) complex (NDUFA2, NDUFA13, NDUFB11, and NDUFS6), the NADH-ubiquinone oxidoreductase (NDUFS1), and the cytochrome c oxidase (NDUFA4).

### Mitochondrial Biogenesis

**Figure 4:**
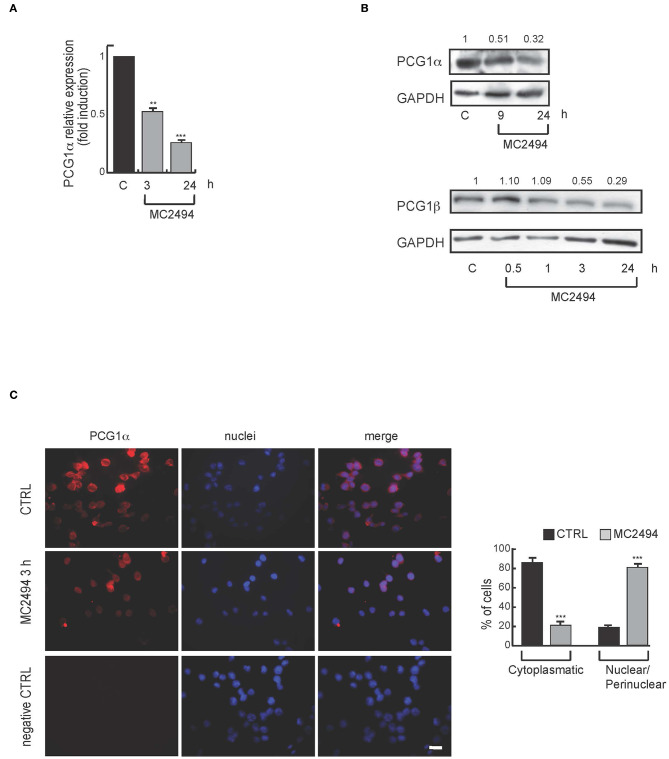
**(A)** mRNA evaluation of PGC1α in U937 cells treated with MC2494 at 50 μM concentration for 3 and 24 h. U937 cells were treated with MC2494 for the indicated times at 50 μM concentration. GAPDH was used as control for equal loading. Graphs show the mean of at least 2 independent experiments with error bars indicating standard deviation. **(B)** *Top panel*, western blot analysis of PGC1α; *bottom panel* western blot analysis of PGC1β. U937 cells were treated with MC2494 at 50 μM concentration for indicated times. Numbers on Western blot indicate the results of densitometry analysis, performed using the Image J Gel Analysis tool. **(C)** Immunofluorescence for PGC1α. Bar, 10 μM. Immunofluorescence microscopy analysis (right graph) is representative of 3 independent experiments. Values are mean ± standard deviation (*SD*) of biological triplicates. ^***^p ≤ 0.001, ^**^p ≤ 0.01 vs. control cells.

Based on proteomic results and given that mitochondria are the major sites where MTT and WST-1 are reduced to formazan product, we further investigated mitochondrial biogenesis after MC2494 treatment. The peroxisome proliferator-activated receptor γ coactivator 1 (PGC1) family has an important function in regulating mitochondrial metabolism (21). We studied the expression of PGC1α, a well-known family member, which is involved in mitochondrial biogenesis and respiration (22). Through transcription of coactivator PGC1α, cancer cells enhance OXPHOS, mitochondrial biogenesis, and oxygen consumption rate (13). In addition, ATP production and energy homeostasis are promoted by PGC1α, generating cells resistant to necrosis and apoptosis (22). Interestingly, in U937 cells we observed a significant and consistent decrease in mRNA levels already after 3 h of MC2494 treatment (Figure 4A). In line with this result, Western blot analysis of PGC1α and PGC1β, both members of the PGC1 family, showed a similar decrease in protein expression with MC2494 treatment at 50 μM for the indicated times (Figure 4B). Immunofluorescence analysis corroborated the observed reduction in PGC1α. After 3 h of MC2494 treatment, immunolabeling of a large number of cells was markedly less intense than that of control cells, confirming a lower expression of PGC1α (Figure 4C and Supplementary Figure 4). Immunofluorescence analysis also revealed that PGC1α was localized mostly (82.6%) in the cytoplasm of control cells. Following treatment with MC2494, the antibody detected lower levels of PGC1α, which had translocated to a greater extent to the perinuclear/nuclear compartment of the cells (Figure 4C and Supplementary Figure 4). These data suggest that in response to MC2494, PGC1α subcellular distribution shifts from the cytoplasm to the perinuclear/nuclear compartment, where it regulates mitochondrial (dys) function.

### MC2494 Alters Mitochondrial Content

**Figure 5:**
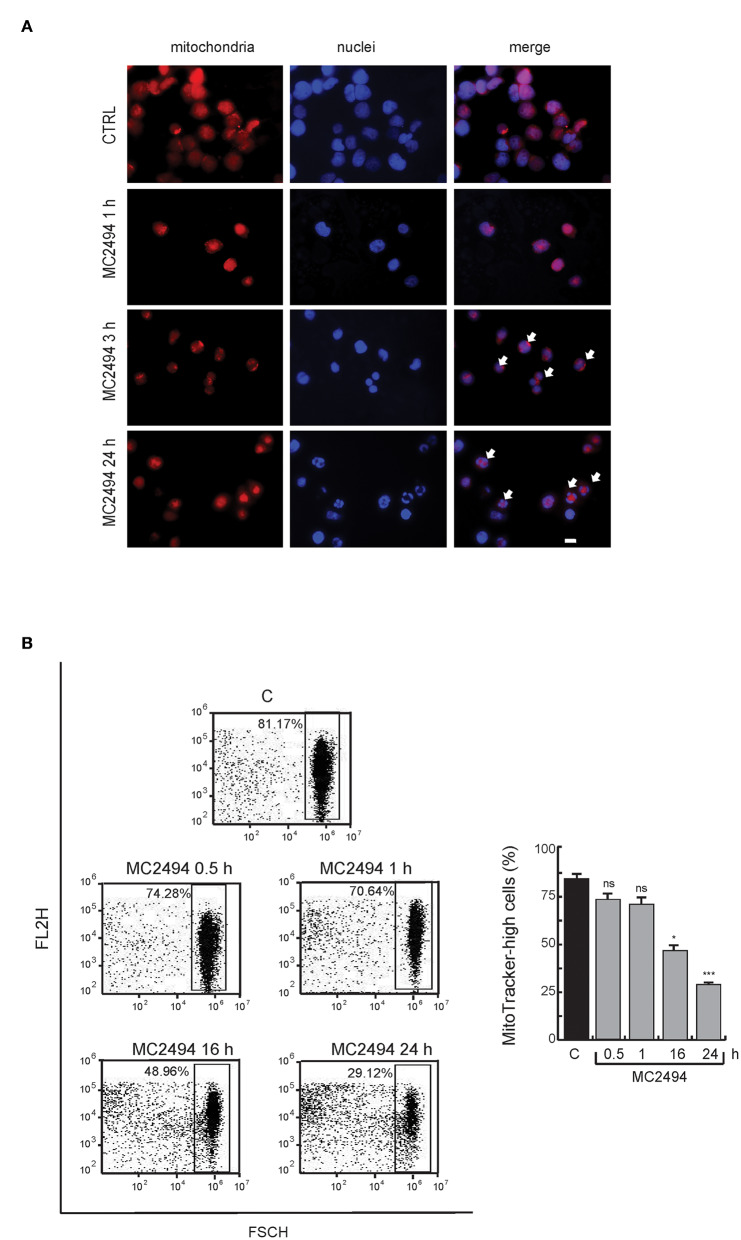
**(A)** Immunofluorescence analysis. MitoTracker Orange staining for cells untreated or treated for the indicated times with MC2494. Images representative of 3 different experiments captured by immunofluorescence microscope show staining of mitochondria (red) and nuclei (blue). Bar, 10 μM. Arrows denote the presence of fragmented mitochondria and nuclei after 3 and 16 h of MC2494 treatment. **(B)** FACS analysis of mitochondrial content. FACS dot plot (left panel) shows a decrease in MitoTracker Orange signal upon MC2494 treatment. Histogram showing data obtained (right panel). Values are mean ± standard deviation (SD) of biological triplicates. ^***^p ≤ 0.001, ^*^p ≤ 0.05, ^ns^*p* > 0.05 vs. control cells.

To assess whether the reduction in PGC1α/β levels coincides with a loss of mitochondria, we studied the total mitochondrial content in U937 cells after MC2494 treatment. In control cells, immunofluorescence microscopy revealed the presence of intact mitochondria mostly localized in the cytoplasm (Figure 5A). Already after 3 h of MC2494 induction, and more evident after 16 h of treatment, an accumulation of fragmented short mitochondria resulting from mitochondrial dysfunction caused by MC2494-induced cell death was observed (Figure 5A). The total disruption of mitochondria consistent with a higher percentage of MC2494-mediated cell death was detected after 24 h of induction (data not shown). We further investigated mitochondrial content by fluorescence-activated cell sorting (FACS). FACS analysis strengthened and confirmed immunofluorescence data, showing an ~30% reduction in mitochondrial content already after 16 h of treatment (Figure 5B).

### MC2494 Induces Mitochondrial Oxidative Stress and ATP Depletion

**Figure 6:**
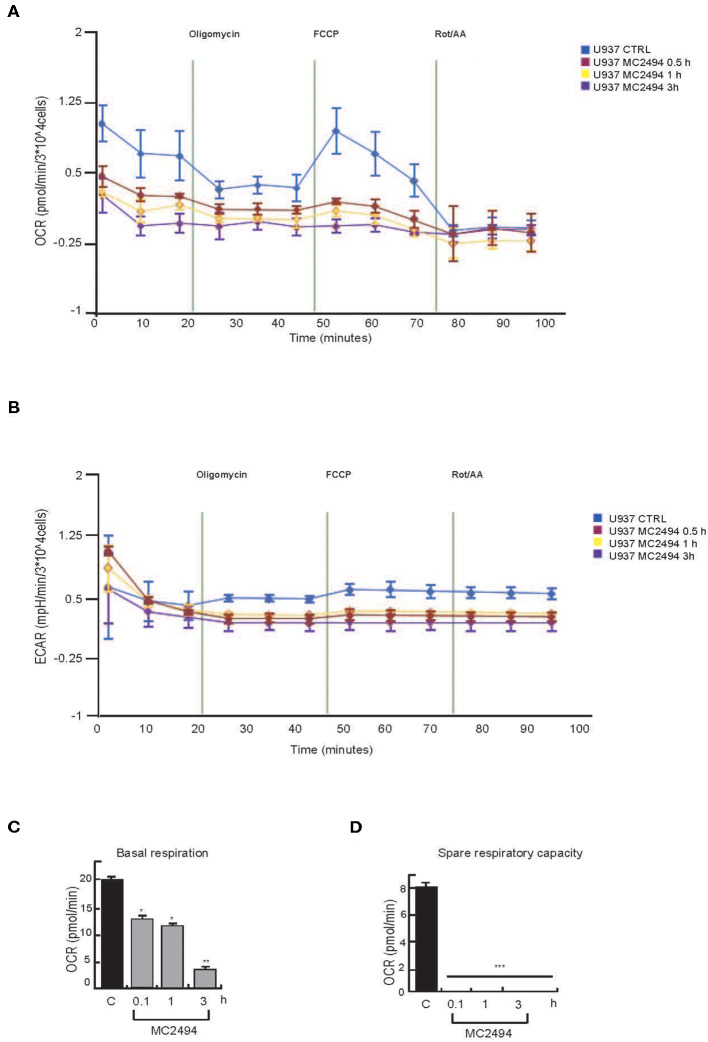
Seahorse analysis. **(A)** OCR evaluation. **(B)** ECAR evaluation. **(C)** Basal respiration evaluation. **(D)** Spare respiratory capacity evaluation. U937 were treated with MC2494 at 50 μM concentration for indicated times. Values are mean ± standard deviation (*SD*) of biological triplicates. ^***^p ≤ 0.001, ^**^p ≤ 0.01, ^*^p ≤ 0.05 vs. control cells.

Given the key role of respiratory electron transport in energy metabolism, which also affects cancer-related processes, we evaluated the effects of MC2494 treatment on mitochondrial function. The decrease in mitochondrial content suggested overall mitochondrial dysfunction and indicated that MC2494 treatment might cause damage to glycolytic capacity and oxidative energy metabolism. To investigate this hypothesis, the OCR and ECAR of live U937 cells were determined in real time. MC2494 treatment at 25 μM concentration caused significant time-dependent mitochondrial dysfunction (Figure 6). The overall metabolic profiles of OCR and ECAR showed a more severe defect of mitochondrial respiration and glycolytic capacity in U937 cells already at the early treatment time (0.5 h, Figures 6A,B). In addition, cell energy metabolism as determined by OCR/ECAR ratio revealed a change from energetic (in basal conditions) to stressed glycolytic quiescent phenotype in a time-dependent manner following MC2494 treatment. In line with these results, basal respiration, and spare respiratory capacity, a known indicator of cell death resistance, were drastically reduced at the same time point of MC2494 treatment (Figures 6C,D).

A comparative study of mitochondrial dysfunction performed in MCF7 cancer cells confirmed all data observed (Supplementary Figure 5).

**Figure 7:**
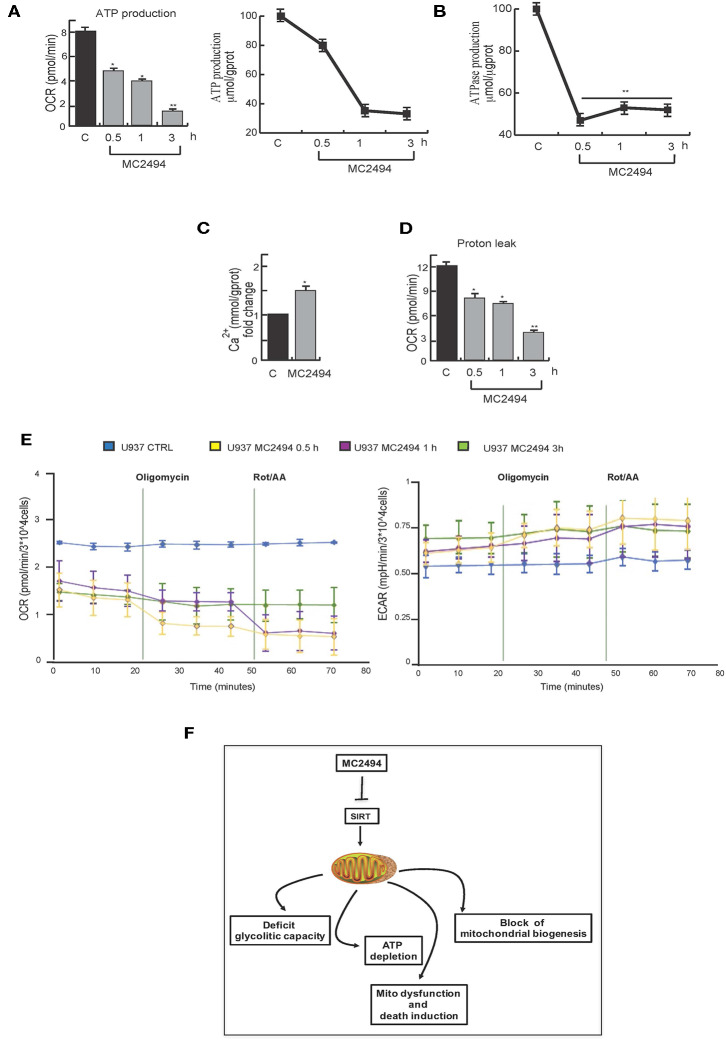
**(A)** Orthogonal ATP assays. **(B)** ATPase assay. U937 were treated with MC2494 at 25 μM concentration for indicated times. **(C)** Intracellular calcium assay. U937 were treated with MC2494 at 25 μM concentration for 24 h. **(D)** Proton leak evaluation. **(E)** Seahorse ATP production assay, OCR and ECAR evaluation. U937 were treated with MC2494 at 25 μM concentration for indicated times. Values are mean ± standard deviation (*SD*) of biological triplicates. ^**^p ≤ 0.01, ^*^p ≤ 0.05 vs. control cells. **(F)** Schematic representation of MC2494 effects on mitochondrial biological activity.

Since the generation of ATP through OXPHOS is one of most important mitochondrial functions, we investigated the effect of MC2494 induction on cellular ATP levels. Two orthogonal *in vitro* assays revealed a strong decrease in ATP production already at the early time of MC2494 treatment (0.5 h, Figure 7A). ATPase activity was also evaluated by *in vitro* assay. As expected, already at early time of induction we observed a reduction in phosphate consumption, which occurs in the exchange of mitochondrial ATP with cytosolic adenosine diphosphate (ADP) (Figure 7B).

To corroborate and strengthen these findings, we measured the rate of ATP production from two different key energy pathways glycolysis capacity and oxidative stress by Seahorse analysis. The observed modulation in OCR and ECAR confirmed robust ATP depletion following MC2494 treatment (Figure 7E). Moreover, as result of mitochondrial dysfunction, several metabolites and molecules are release, among them Ca^2+^. Following treatment with MC2494 at 25 μM for 24 h, we observed an increment of cytosolic Ca^2+^, as intra- and extra- messenger of several cellular pathways from cell proliferation to cell death (Figure 7C).

Several studies report that acidification of the cytosol occurs as a consequence of cell death pathway induction (23). In response to MC2494 treatment, after 3 h of challenging, we observed a strong proton leak of mitochondria, which may contribute to activating MC2494-mediated cell death pathways (Figure 7D).

## Discussion

Mitochondria are organelles that play a key role in cellular biology and disease due to their involvement in different processes, including bioenergetics, apoptosis, and cell signaling. Modulation of mitochondrial protein expression and function also regulate cellular metabolic homeostasis, whose dysregulation is frequently observed in several tumors (24).

Induction of mitochondrial damage is proposed as an alternative mechanism for inhibiting tumor growth (25, 26). Some reports correlate increased ATP production with tumor cell growth, underscoring the involvement of mitochondria in the development of chemotherapy resistance (27, 28).

Although the role of mitochondria and the involvement of mitochondrial SIRTs in apoptosis are well-described, very little is known about the molecular mechanism underlying the SIRT-mediated modulation of this process.

Furthermore, it is widely reported that some SIRTs located in mitochondria may exert critical functions as regulators of OXPHOS, directly affecting mitochondrial enzymes (29).

We previously demonstrated that the pan-SIRT inhibitor MC2494 causes an increase in ROS production, a consistent dissipation of MMP, and activation of mitochondrial apoptotic pathway (17).

In the present study, we investigated the effects of MC2494 treatment on mitochondria and identified potential connections between MC2494-mediated cellular response and mitochondrial dysfunction.

The relative complexity of the mitochondrial proteome requires the use of high-throughput methodologies for the large-scale profiling of key pathways involved in mitochondrial dysfunction. In an effort to gain a greater understanding of the molecular effects of MC2494 on mitochondrial proteome, we adopted an advanced quantitative proteomic approach based on TMT isobaric labeling coupled with nanoLC-MS/MS. By performing extensive profiling of mitochondrial proteins in MC2494-treated and untreated U937 cells, we comprehensively mapped proteins relevant to mitochondrial functions and identified a specific subset of down-regulated proteins specifically involved in respiratory electron transport and associated pathways.

Growing scientific interest is focusing on mitochondrial function in terms of the key role of energy homeostasis in cell viability, and mitochondria biogenesis. Higher mitochondrial mass and increased synthesis of specific metabolic enzymes are necessary to meet increased ATP demand during cell proliferation. First, by treating U937 cells with MC2494 we confirmed its well-known anti-proliferative effects. In line with our previously published results (17), evaluation of formazan reduction together with induction of SOD2 expression revealed a strong inhibition of cell proliferation, highlighting the involvement of mitochondria in this process. Mechanistically, upon MC2494 treatment we observed a reduction in some important mitochondrial biogenesis proteins at both mRNA and protein level, suggesting a reduction in mitochondria mass. These findings were confirmed by immunofluorescence and FACS analysis.

The observed differential expression of enzymes with key roles in mitochondrial energy metabolism supports the use of proteomics-based approaches to complement gene expression analyses in order to highlight different levels of post-transcriptional regulation. The selective down-regulation of core constituents of the OXPHOS system, essential for ATP synthesis and energy metabolism homeostasis, also led us to further investigate the effects of MC2494 treatment on mitochondrial function. The MC2494-mediated inhibition of mitochondrial biogenesis had an impact on respiratory capacity and ATP production, determining a strong decrease in both at the early treatment time. Changes in ATP:ADP ratio due to the fall in ATP production reflecting the decrease in respiratory capacity strongly indicate a general mitochondria dysfunction. Given that most cancers rely on anaerobic glycolysis for ATP production, the block of mitochondrial function mediated by MC2494 may play a pivotal role in several tumors modulating redox and energy homeostasis, transcriptional regulation, and cell death. p53 loss-of-function, occurs in AML, following its mutation/deletion (30) or by overexpression of its negative regulator Mdm2 (31, 32) and is associated with a low survival rate. Moreover, Mdm2 interfers with the recruitment of several acetyltransferases (33) blocking p53 acetylation and its transcriptional activity. Elevated sirtuins expression has been reported in AML and in other cancers (34) and promotes tumourigenesis and drug resistance (6). Particularly, SIRT1 deacetylates different histone and non-histone proteins, among them p53, regulating both cell cycle and mitochondrial function (35) and playing a critical role in tumor initiation progression and metastasis (30, 36, 37). Effects of p53 on regulation of PGC1α, a metabolic modulator in cancer (38, 39) have been previously reported (40, 41), with p53-mediated PGC1α up-regulation orchestrating antioxidant and metabolic response (42). Since U937 cell line lacks of p53 (43), our results shed light on other key molecular players driven MC2494 anticancer property mediated by mitochondrial dysfunction.

However, that in other human myeloid leukemia cells expressing WT p53, SIRT1 may mediate p53-PGC1α regulation axis also by the interaction with other proteins such as RIP1 (17) cannot be excluded. Further investigations are required to better characterize in these cell systems, the role of p53 in MC494-induced signaling pathways. In these circumstances, mitochondria are promising targets for the development of anticancer therapies. Our findings provide an insight into how the novel SIRT inhibitor MC2494 modulates mitochondria by controlling their biogenesis and determining their dysfunction (Figure 7F). Proteomic approaches could provide a valuable tool to advance our understanding of the effects of other SIRT inhibitors and to identify molecular determinants affected by treatment with these compounds. Taken together, our findings strengthen the concept that mitochondrial damage can be considered an anticancer mechanism and might represent an attractive target in therapeutic strategies.

## Data Availability Statement

The original contributions presented in the study are publicly available. This data can be found here: the ProteomeXchange Consortium via the PRIDE partner repository with the dataset identifier PXD017508 (https://www.ebi.ac.uk/pride/archive/projects/PXD017508).

## Author Contributions

VC and RR: conceptualization. VC, RR, LD, FC, FS, CD, and GS: main experiments. MD: immunofluorescence experiments. DR and AM: chemistry. VC, AC, GC, AN, and LA: writing. All authors gave final approval of the manuscript.

## Conflict of Interest

The authors declare that the research was conducted in the absence of any commercial or financial relationships that could be construed as a potential conflict of interest.
