# Supplementary material for: The Pan-Sirtuin Inhibitor MC2494 Regulates Mitochondrial Function in a Leukemia Cell Line
Source: Front Oncol. 2020 May 21;10:820. doi: 10.3389/fonc.2020.00820 (PMC7255067; doi:10.3389/fonc.2020.00820)
Supplement: Supplementary file 1 [file Image_1.pdf]

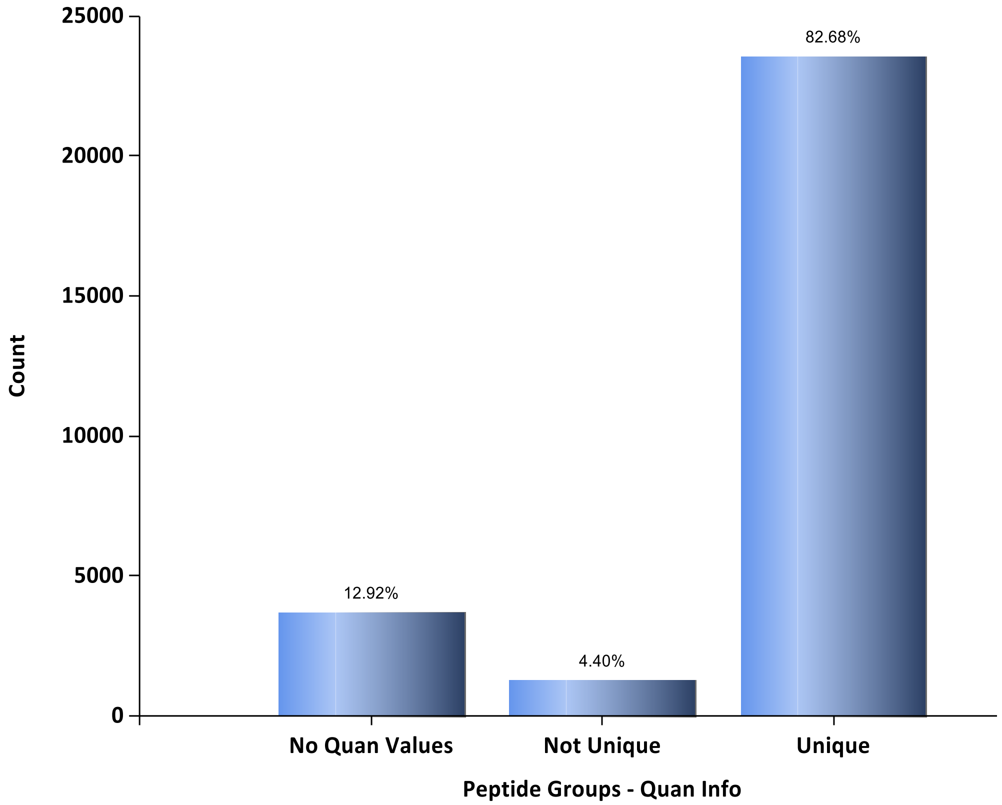

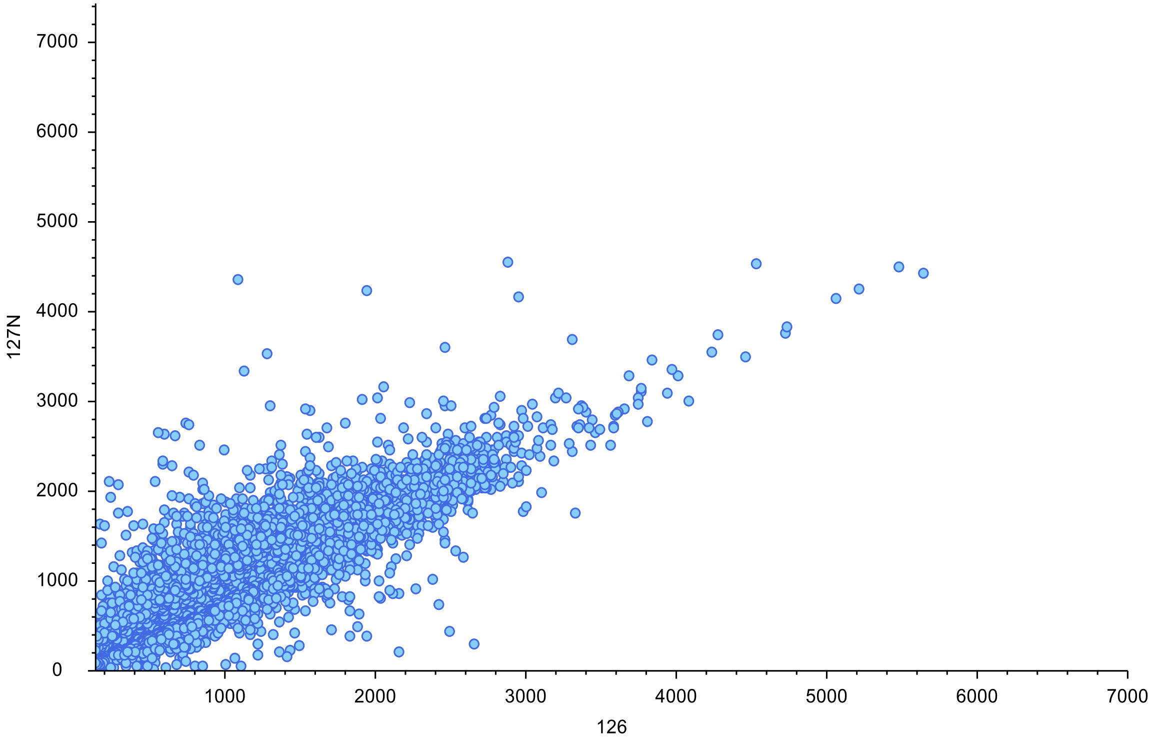

Supplementary figure 1. A) Peptide group distribution used for protein quantification. A total of 82.7% of unique peptides was used for protein quantification, guaranteeing the high efficiency of peptide labeling. B) Scatter plot of peptide intensities in untreated (127N TMT tag) *versus* MC2494-treated (126 TMT tag) U937 cells.
