# Supplementary material for: The Pan-Sirtuin Inhibitor MC2494 Regulates Mitochondrial Function in a Leukemia Cell Line
Source: Front Oncol. 2020 May 21;10:820. doi: 10.3389/fonc.2020.00820 (PMC7255067; doi:10.3389/fonc.2020.00820)
Supplement: Supplementary file 2 [file Image_2.pdf]

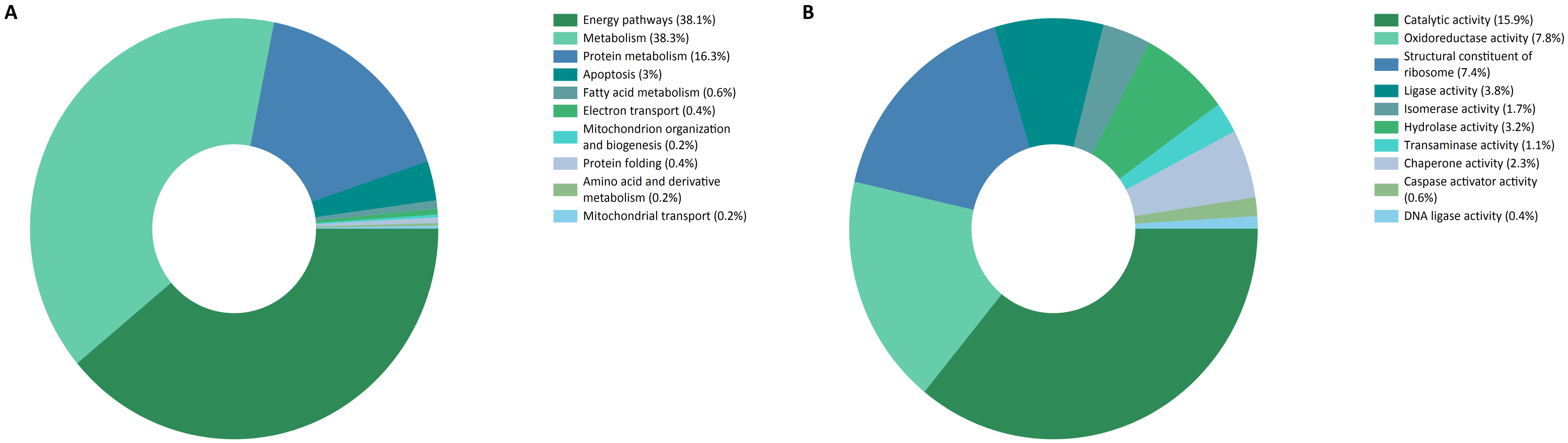

Supplementary figure 2. Functional enrichment based on Gene Ontology (GO) categories of mitochondrial proteins identified by nanoLC-MS/MS analysis. A) Enrichment analysis performed for the biological process GO category revealed a significant enrichment of proteins involved in energy pathways, metabolism/protein metabolism, and apoptosis. B) Enrichment analysis for the molecular function GO category revealed a significant enrichment of proteins involved in catalytic and oxidoreductase activity. Enrichment analysis was based on a hypergeometric test using FunRich software.
