# Supplementary material for: The Pan-Sirtuin Inhibitor MC2494 Regulates Mitochondrial Function in a Leukemia Cell Line
Source: Front Oncol. 2020 May 21;10:820. doi: 10.3389/fonc.2020.00820 (PMC7255067; doi:10.3389/fonc.2020.00820)
Supplement: Supplementary file 3 [file Image_3.pdf]

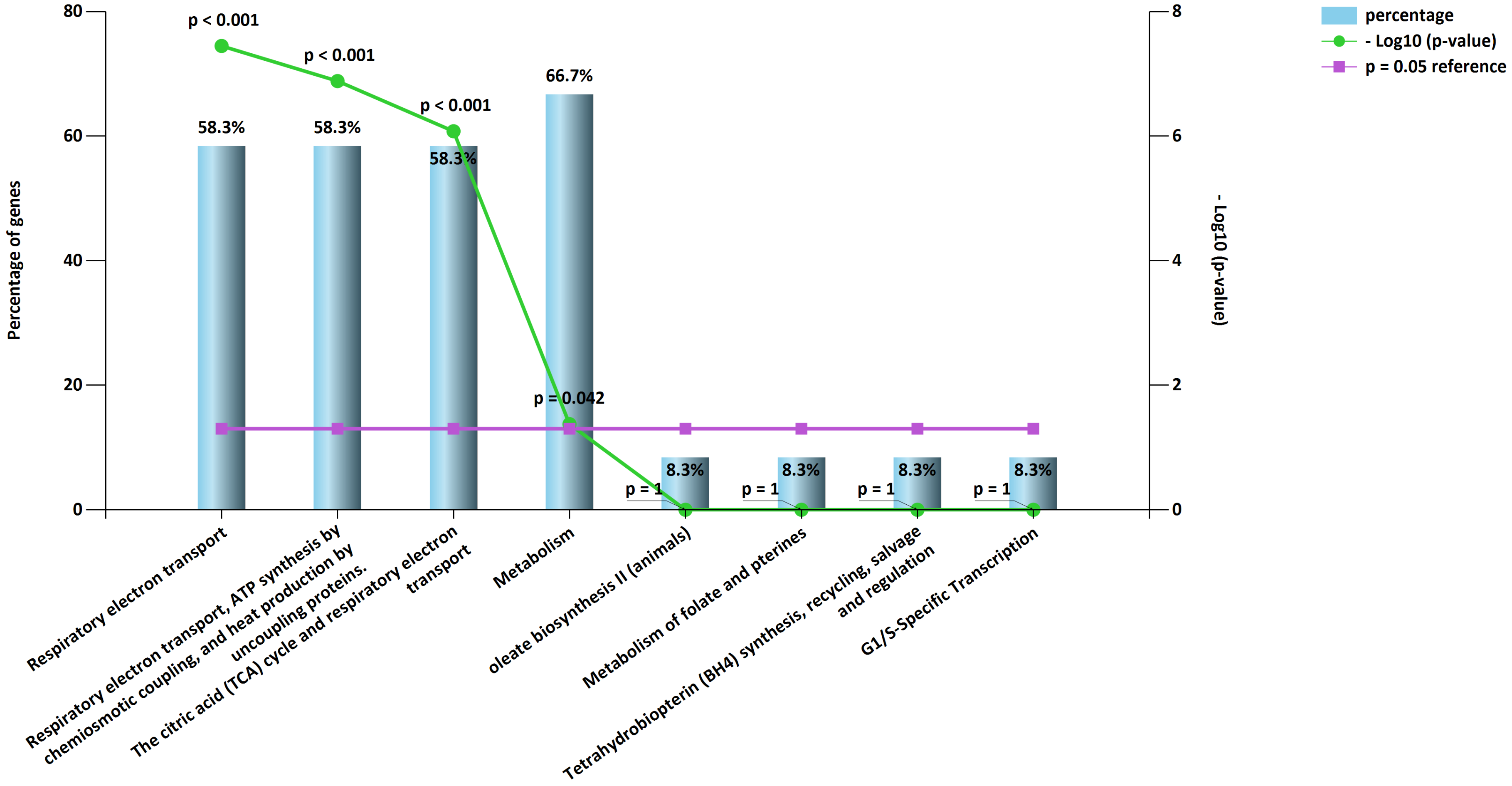

Supplementary figure 3. Functional enrichment based on the biological process GO category of differentially expressed mitochondrial proteins identified by TMT-based quantitative proteomic analysis of MC2494-treated *versus* untreated U937 cells. Enrichment analysis was performed using FunRich software. Percentages of mapped proteins/genes and p-values based on a hypergeometric test are reported in bar graphs along with reference p-value (p = 0.05).

Carafa et al. Supplementary Figure 3
