# Supplementary material for: The Pan-Sirtuin Inhibitor MC2494 Regulates Mitochondrial Function in a Leukemia Cell Line
Source: Front Oncol. 2020 May 21;10:820. doi: 10.3389/fonc.2020.00820 (PMC7255067; doi:10.3389/fonc.2020.00820)
Supplement: Supplementary file 4 [file Image_4.pdf]

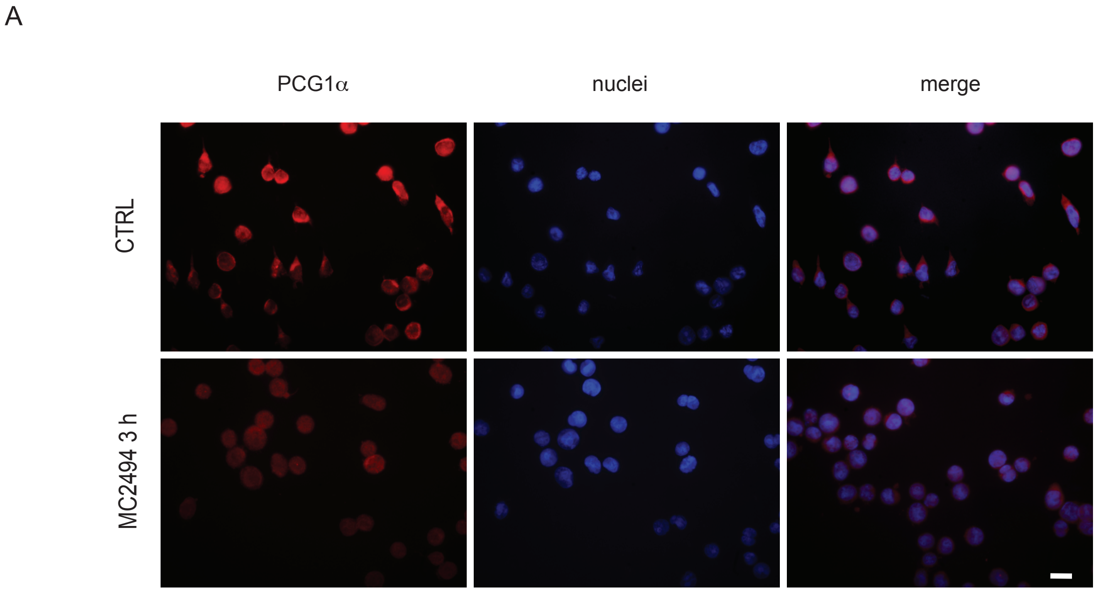

Supplementary figure 4. Immunofluorescence for PGC1α. Bar, 10 μM. Fields were acquired using a DMBL Leica fluorescence microscope equipped with HCX PL Fluotar Apo 63X oil objectives
