# Supplementary material for: The Pan-Sirtuin Inhibitor MC2494 Regulates Mitochondrial Function in a Leukemia Cell Line
Source: Front Oncol. 2020 May 21;10:820. doi: 10.3389/fonc.2020.00820 (PMC7255067; doi:10.3389/fonc.2020.00820)
Supplement: Supplementary file 5 [file Image_5.pdf]

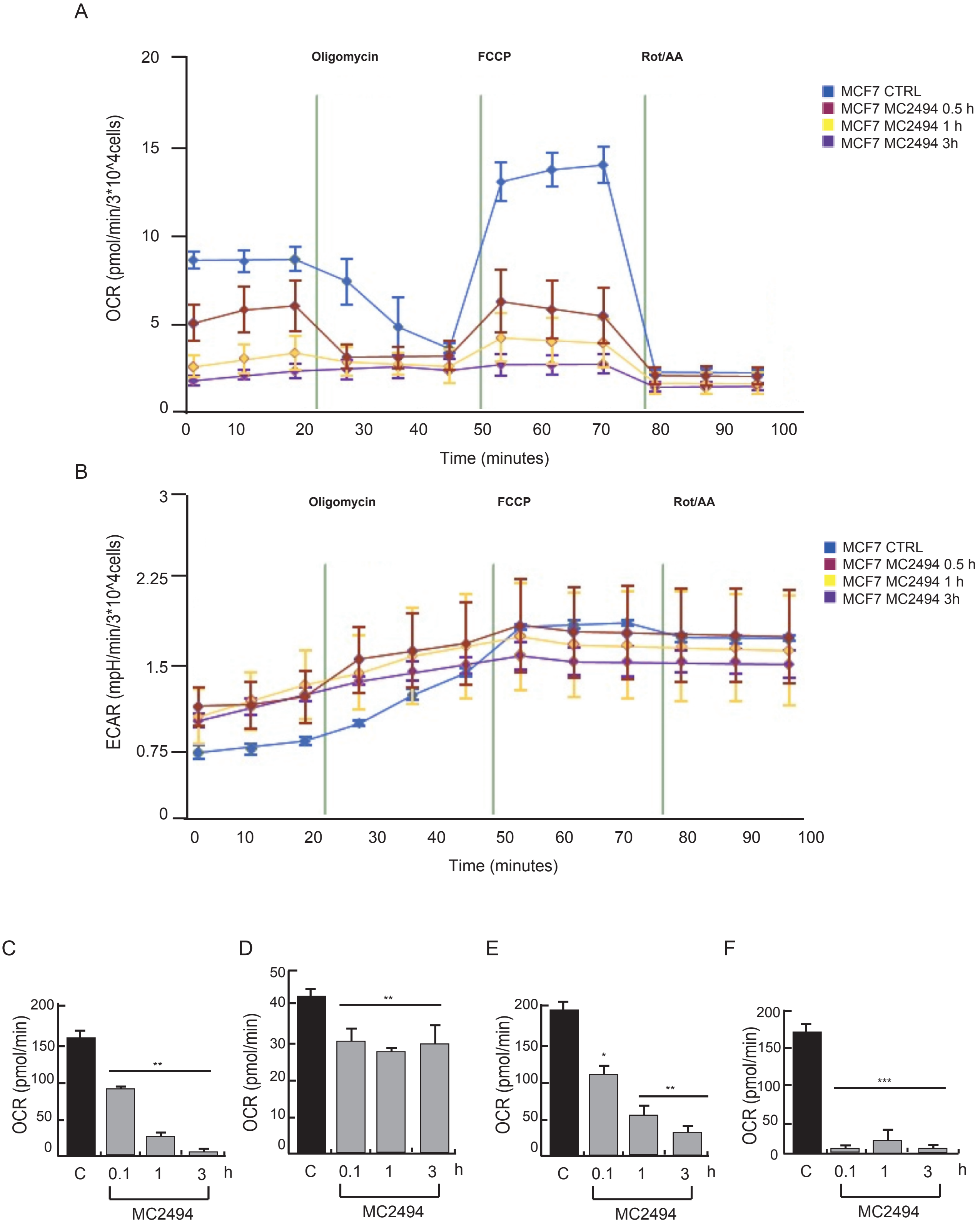

Supplementary figure 5. Seahorse analysis. A) OCR evaluation. B) ECAR evaluation. C) Basal respiration evaluation. D) Spare respiratory capacity evaluation. E) ATP evaluation. F) Proton leak evaluation. MCF7 were treated with MC2494 at 50 μM concentration for indicated times.
Values are mean ± standard deviation (SD) of biological triplicates. ***p-value ≤ 0.001, **p-value ≤ 0.01, *p-value ≤ 0.05 vs. control cells.
